# The diabetic shoulder: association between diabetes mellitus and adhesive capsulitis — a systematic review and meta-analysis

**DOI:** 10.1007/s00264-026-06793-4

**Published:** 2026-03-28

**Authors:** Philippe Hernigou, Marius M. SCARLAT

**Affiliations:** 1 Paris-Est Créteil University, Créteil, France; 2 Orthopaedic Department, Clinique Chirurgicale St Michel, Groupe ELSAN, Toulon, 83100 France

**Keywords:** Diabetic shoulder, Diabetes Mellitus, Adhesive capsulitis, Frozen shoulder, Shoulder stiffness

## Abstract

**Purpose:**

To assess whether diabetes mellitus (type 1 or type 2) increases the risk of developing adhesive capsulitis (frozen shoulder).

**Methods:**

A systematic review and meta-analysis were conducted in 2026 using multiple electronic databases. Additional studies were identified through screening reference lists and consulting professional networks. Studies examining the relationship between diabetes mellitus and the incidence of adhesive capsulitis were eligible for inclusion. Study quality and bias risk were assessed, and when enough data were available, a random-effects meta-analysis was performed to estimate the general association between diabetes and the development of frozen shoulder.

**Results:**

The combined analysis showed that people with diabetes had 3.69 times higher odds (95% CI 2.99–4.56) of developing adhesive capsulitis compared to people without diabetes. Several additional risk factors were noted across studies, including poor glycemic control, obesity, hyperlipidaemia, hypertension, thyroid issues, age between 40 and 65, female gender, smoking, and alcohol use.

**Conclusion:**

Diabetes mellitus is strongly associated with an increased risk of adhesive capsulitis. However, the observed association may be influenced by unmeasured confounding factors. Further high-quality longitudinal studies are required to clarify the causal relationship and underlying mechanisms.

Although the association between diabetes mellitus and frozen shoulder was first described more than 25 years ago by Scarlat and Harryman [1], the relationship remains incompletely understood and remains under debate among orthopaedic surgeons. As a result, diagnosis and management of this condition may be delayed, potentially leading to prolonged disability. The purpose of this review is to examine the association between diabetes mellitus and adhesive capsulitis and to identify factors that may increase the risk of frozen shoulder in diabetic patients.

Diabetes mellitus (DM) is one of the most prevalent chronic diseases worldwide, and its incidence continues to rise steadily. It is estimated that more than 530 million adults currently live with diabetes, a number projected to increase to approximately 642 million by 2040 [1–3]. While the cardiovascular, renal, and neurological complications of diabetes are well recognized, musculoskeletal manifestations remain underappreciated despite their substantial impact on morbidity and quality of life.

The concept of the “diabetic shoulder” mainly refers to adhesive capsulitis (AC), also known as frozen shoulder. This condition is characterized by shoulder pain and progressive restriction of both active and passive range of motion, particularly external rotation. Numerous epidemiological studies and meta-analyses have reported a strong association between diabetes and adhesive capsulitis [4–8].

Several pathophysiological mechanisms have been proposed to explain this association. Chronic hyperglycaemia may promote the formation of advanced glycation end products (AGEs), which alter collagen structure and lead to capsular fibrosis and contracture. In addition, inflammatory processes and microvascular changes associated with diabetes may further contribute to the development of capsular stiffness [8].

To better understand the relationship between diabetes and adhesive capsulitis, this review synthesizes current evidence on epidemiology, pathophysiology, clinical presentation, diagnostic considerations, and management strategies. In addition, we examine other potential risk factors associated with frozen shoulder and discuss implications for clinical practice and future research.

## Methods

### Literature search strategy

**Fig. 1:**
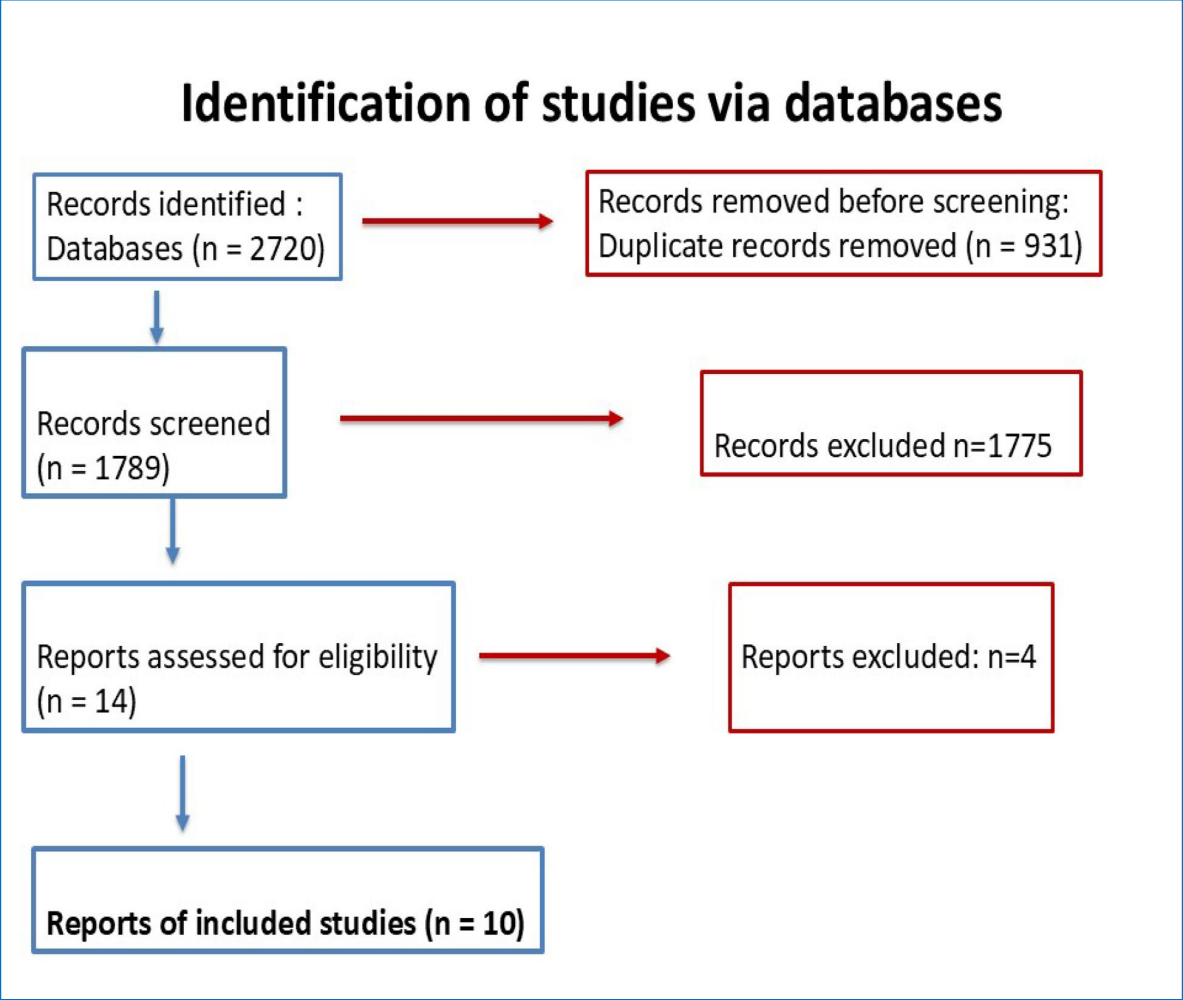
Flow Diagram

The systematic review was conducted according to the Preferred Reporting Items for Systematic Reviews and Meta-Analyses (PRISMA) guidelines [9], and a PRISMA flow diagram summarizing the study selection process is presented in Fig. 1.

A comprehensive literature search was performed in 2026 using MEDLINE, Embase, and Google Scholar. Reference lists of relevant articles were also screened to identify additional eligible studies. The search strategy included both controlled vocabulary terms (e.g., Medical Subject Headings) and free-text keywords related to diabetes mellitus and shoulder disorders.

A comprehensive literature search was conducted in MEDLINE (via PubMed), Embase, and Google Scholar from January 2000 to December 2025. The search strategy combined controlled vocabulary (MeSH terms) and free-text keywords related to diabetes mellitus and adhesive capsulitis. The following search terms were used: ("diabetes mellitus" OR "type 1 diabetes" OR "type 2 diabetes") AND ("adhesive capsulitis" OR "frozen shoulder" OR "shoulder stiffness"). Reference lists of included studies and relevant reviews were also screened to identify additional eligible studies.

### Study selection

All titles and abstracts identified through the literature search were screened independently to determine eligibility according to predefined inclusion and exclusion criteria. Studies published between 2000 and 2025 were considered for inclusion.

To qualify for inclusion, studies were required to have a longitudinal observational design, either prospective or retrospective. Cohort studies were eligible if they included participants without adhesive capsulitis at baseline and documented the presence of diabetes mellitus. Case–control studies were included if they compared individuals diagnosed with adhesive capsulitis with a control group without the condition, with diabetes mellitus as the primary exposure variable.

There were no restrictions regarding study setting, and both population-based studies and hospital-based clinical cohorts were considered eligible. The following types of studies were excluded: cross-sectional studies; case series; studies without accessible full text; non-English language publications.

Studies that did not clearly define the diagnosis of adhesive capsulitis or diabetes mellitus were also excluded.

### Study characteristics

The literature search identified 1,789 unique citations, of which 14 articles were selected for full-text screening. After applying the eligibility criteria (Fig. 1), ten studies [10–20] were included in the final analysis, representing a total population of 351,486 participants. Among these studies, seven were case–control studies, three were cohort studies. The proportion of female participants ranged from 51 to 76%, and the mean age ranged from 50 to 70 years. Only two studies were published in orthopaedic surgery journals, whereas the remaining studies originated from broader medical or epidemiological research fields.

### Data extraction

Data extraction was performed using a standardized form. The following information was collected from each study: study design and setting, sample characteristics, method used to diagnose diabetes mellitus, method used to diagnose adhesive capsulitis, inclusion and exclusion criteria, sample size, duration of follow-up, covariates included in statistical models, statistical analyses performed, measures of association (odds ratio, hazard ratio, or risk ratio).

When studies reported both adjusted and unadjusted estimates, the adjusted estimates were preferentially extracted. If effect estimates were not directly reported, available data were used to calculate the corresponding association measures when possible. The eight most important studies are reported in Table 1.

**Table 1:** Characteristics of Included Studies

Study	Country	Study Design	Sample Size	Mean Age (years)	% Female	Diagnosis of Adhesive Capsulitis	Diabetes Assessment	Main Adjusted Variables
Boyle-Walke et al	USA	Case–control	32 cases	NR	75%	Clinical examination	Medical history	Sex
Li et al	China	Case–control	182 cases	57.2	63%	Clinical diagnosis	Fasting glucose/HbA1c	Not reported
Lee et al	Korea	Case–control	40 cases	52.8	55%	MRI + clinical criteria	Laboratory diagnosis	Age, sex
Milgrom et al	Israel	Case–control	126 cases	55	60%	Clinical diagnosis	Blood glucose > 200 mg/dL	Age
Wang et al	Australia	Case–control	87 cases	56	64%	Clinical examination	Medical records	Age, sex
Kingston et al	USA	Case–control	2,190 cases	56.4	58%	Administrative coding	Medication records	Sex
Huang et al	Taiwan	Cohort	78,827	55.7	47%	National health database codes	Medical records	Age, sex, dyslipidemia
Lo et al	Taiwan	Cohort	5,109	NR	52%	National health database codes	Medical records	Age, income, hypertension, hyperlipidemia
Park et al	Korea	Case–control	210	NR	NR	Clinical diagnosis	Laboratory data	BMI, age
Dyer et al	UK	Systematic review	5,388	NR	NR	Mixed definitions	Mixed definitions	Various

*NR* = Not reported

### Risk of bias assessment

The methodological quality of the included studies was evaluated using the Quality in Prognosis Studies (QUIPS) tool, which assesses risk of bias across several domains including: study participation; study attrition; measurement of exposure; measurement of outcomes; confounding factors; statistical analysis and reporting. Each study was classified as having low, moderate, or high risk of bias based on the overall assessment across these domains. Adhesive capsulitis was defined according to the diagnostic criteria used in each included study. In most studies, diagnosis was based on clinical assessment of shoulder pain associated with restriction of both active and passive range of motion, particularly external rotation. Some studies relied on administrative diagnostic codes from healthcare databases.

Statistical heterogeneity between studies was assessed using the I^2^ statistic and Cochran’s Q test. Heterogeneity was interpreted as follows: low heterogeneity: I^2^ < 25%; moderate heterogeneity: I^2^ = 25–50%; substantial heterogeneity: I^2^ > 50%.

### Data analysis

Case–control studies and cohort studies were analyzed separately due to differences in study design and effect measures. For cohort studies, the association between diabetes mellitus and adhesive capsulitis was expressed using hazard ratios (HRs). For case–control studies, associations were expressed using odds ratios (ORs). If fewer than five studies were available for a specific analysis, the results were summarized using narrative synthesis. When five or more studies were available, a random-effects meta-analysis was conducted to estimate the pooled effect size. A random-effects model was selected to account for potential between-study heterogeneity due to differences in study populations, diagnostic criteria, and study design. Forest plots were generated to illustrate individual study estimates and the pooled effect size.

Potential small-study effects and publication bias were visually assessed using funnel plots. However, formal statistical tests for funnel plot asymmetry were not conducted because fewer than ten studies were included in the meta-analysis.

### Search strategy for associated risk factors

In addition to examining the association between diabetes mellitus and adhesive capsulitis, this review also explored other clinical and metabolic factors that may contribute to the development of frozen shoulder. An observational review approach was used to synthesize evidence regarding potential risk factors reported in the literature.

These factors included: metabolic variables, endocrine disorders, demographic characteristics, lifestyle factors.

Because of the heterogeneity of study designs and outcome definitions, the analysis of associated risk factors was primarily conducted using narrative synthesis rather than quantitative meta-analysis.

## Results

### ASSOCIATION BETWEEN DIABETES MELLITUS AND ADHESIVE CAPSULITIS

#### Evidence from observational studies

**Fig. 2:**
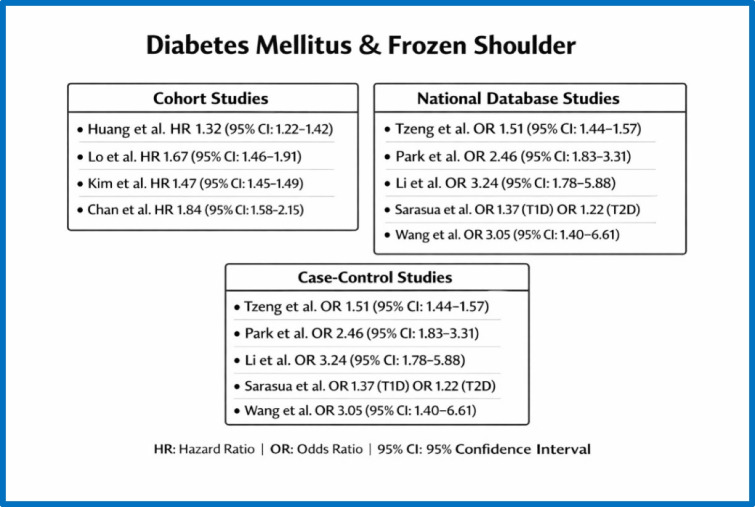
studies analyzing the association between diabetes mellitus and frozen shoulder

A total of eleven observational studies, including four cohort studies and seven case–control studies [10–27], examined the link between diabetes mellitus and adhesive capsulitis. These studies (Fig. 2) consistently reported a higher prevalence or incidence of frozen shoulder among people with diabetes compared to those without the condition.

In cohort studies, diabetes mellitus was linked to a higher risk of developing adhesive capsulitis. Reported adjusted hazard ratios (HRs) ranged from 1.08 for people with prediabetes to 1.67 for those with diabetes. Additionally, higher glycaemic levels correlated with an increased risk of frozen shoulder; for example, HbA1c levels above 7% were linked to an odds ratio (OR) of 1.84 for developing adhesive capsulitis. Moderate heterogeneity was observed across the studies.

Similarly, case–control studies consistently showed a higher prevalence of diabetes among patients with frozen shoulder. Adjusted odds ratios ranged from 1.22 to 3.24, depending on the diagnostic criteria used for diabetes mellitus and adhesive capsulitis. One study that only performed univariate analysis reported an odds ratio of 3.05, further supporting the link between diabetes and frozen shoulder.

Additionally, one case–control study found that the use of diabetes medications was significantly more common among patients with adhesive capsulitis (18.4%; 95% CI, 12.9–25.7) compared to control subjects (7.6%; 95% CI, 6.7–8.5), indicating a higher prevalence of diabetes-related metabolic disturbances in individuals with frozen shoulder.

#### Large population-based studies

Large epidemiological studies utilizing national or regional healthcare databases (Table 2) have also verified the link between diabetes mellitus and adhesive capsulitis. These population-based cohort studies showed that people with diabetes face a three- to fivefold higher risk of developing frozen shoulder compared to those without diabetes.

**Table 2:** Epidemiological studies linking diabetes and adhesive capsulitis

Study	Design	Sample Size	Key Findings
Dyer et al. 2025	cohort	43,977	HR 4.46
Huang et al	Cohort	Large database	HR 1.32
Lo et al	Cohort	National database	HR 1.67
Park et al	Case–control	Clinical sample	OR 2.46
Li et al	Case–control	Hospital-based	OR 3.24

For example, a large series by Dyer et al. [18] reported a hazard ratio (HR) of 4.46 (95% CI: 3.68–5.41), indicating a significantly higher risk of adhesive capsulitis in diabetic populations. A large cohort study by Huang et al. identified diabetes mellitus as an independent risk factor for frozen shoulder, with a hazard ratio of 1.32. Another nationwide cohort study by Lo et al. reported a hazard ratio of 1.67, further supporting the increased risk associated with diabetes.

These large-scale epidemiological studies provide strong evidence that diabetes mellitus represents a major risk factor for adhesive capsulitis.

#### Meta-analysis

**Fig. 3:**
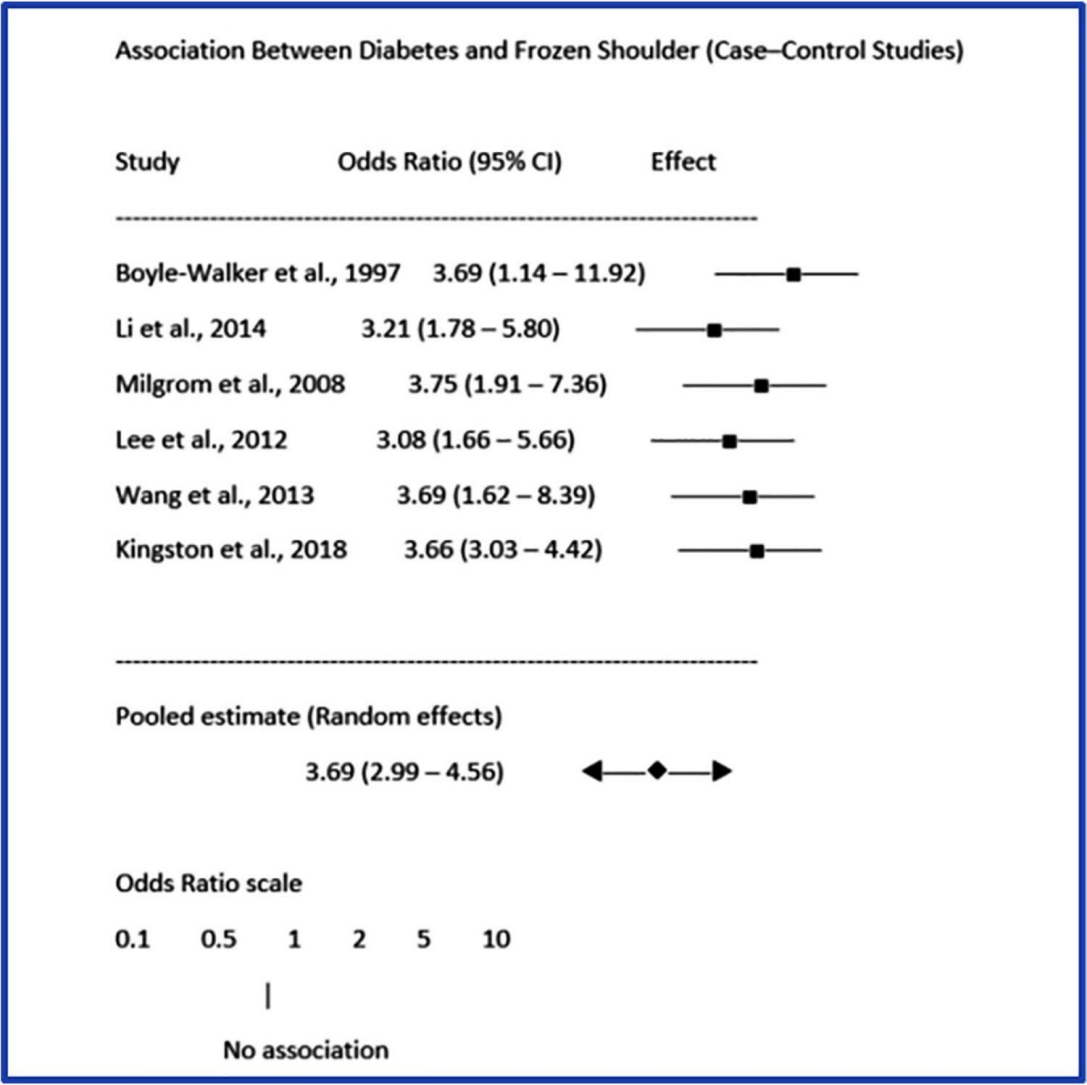
The pooled random-effects estimate shows that people with diabetes have about 3.7 times higher odds of developing frozen shoulder compared with people without diabetes

A meta-analysis was performed using data from six case–control studies examining the link between diabetes mellitus and adhesive capsulitis. The forest plot (Fig. 3) shows the effect estimates for each study along with the combined estimate derived from a random-effects model. In the forest plot, each horizontal line indicates the odds ratio and its 95% confidence interval (CI) for a specific study, while the square marker displays the effect size's point estimate. The size of the square indicates each study's statistical weight in the meta-analysis. All included studies showed odds ratios greater than 1, suggesting a consistent link between diabetes mellitus and a higher risk of developing adhesive capsulitis.

The pooled random-effects model produced an overall odds ratio of 3.69 (95% CI 2.99–4.56), showing that individuals with diabetes have about 3.7 times higher odds of developing frozen shoulder compared to those without diabetes. The generally consistent direction of the effect estimates across studies indicates limited variability among the study populations.

### Assessment of publication bias

**Fig. 4:**
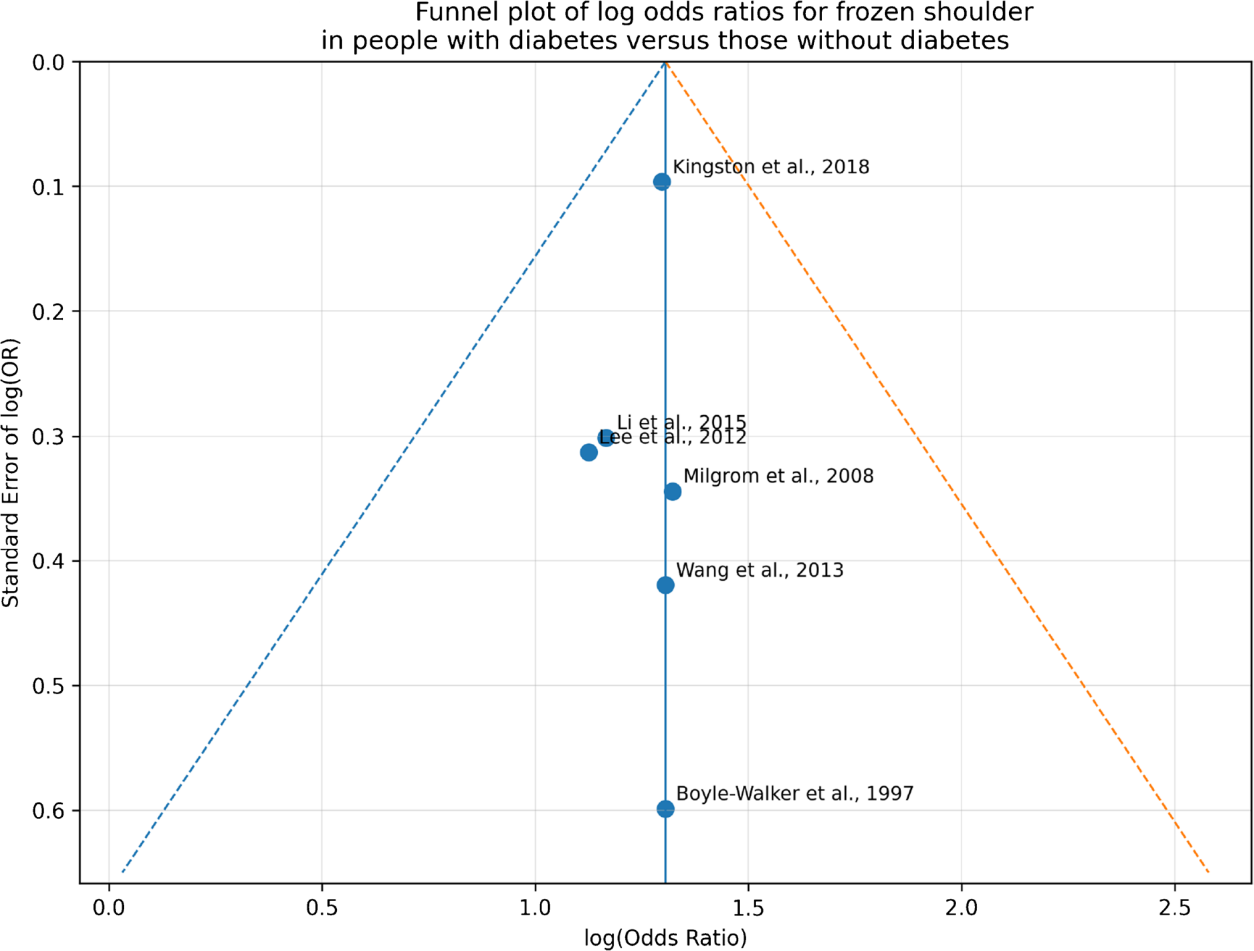
Funnel plot of log ORs for developing frozen shoulder in people with diabetes versus those without diabetes

A funnel plot (Fig. 4) was created to visually evaluate potential small-study effects and publication bias. In this plot, the log of the odds ratios was plotted against their standard errors. Although the distribution of studies seemed mostly symmetrical, a formal statistical test for funnel plot asymmetry (such as Egger’s test) was not conducted because fewer than ten studies were included in the meta-analysis.

Therefore, the presence of publication bias cannot be completely excluded.

Overall, the available evidence shows that: Diabetes mellitus is strongly linked to a higher risk of adhesive capsulitis. Observational studies consistently find a greater prevalence and incidence of frozen shoulder among people with diabetes. The pooled meta-analysis indicates that individuals with diabetes have about three to four times higher odds of developing adhesive capsulitis compared to those without diabetes.

## EPIDEMIOLOGY OF ADHESIVE CAPSULITIS IN DIABETES

### Prevalence and incidence

Adhesive capsulitis is a common shoulder condition marked by increasing pain and limited movement in both active and passive ranges. In the general population [28–32], the estimated prevalence ranges from 2 to 5%, with the highest occurrence between ages 40 and 65 and a slight female predominance. In people with diabetes mellitus, the occurrence of adhesive capsulitis is significantly higher. Epidemiological studies show prevalence rates from 13 to 30%, and in some groups of patients with type 1 diabetes, the prevalence has been reported as high as 76%.

Longitudinal cohort studies further support this association. Analyses based on large primary care databases in the United Kingdom [18] have shown that individuals with type 2 diabetes have a significantly increased risk of developing adhesive capsulitis compared with non-diabetic individuals. In these studies, type 2 diabetes was associated with a hazard ratio of approximately 4.38 for the development of frozen shoulder.

The findings of the present meta-analysis are consistent with these observations, indicating that diabetes mellitus increases the risk of adhesive capsulitis by approximately three to four times compared with the general population. The pooled prevalence of frozen shoulder in diabetic populations has been estimated at approximately 13.4%.

### Type 1 versus type 2 diabetes

**Fig. 5:**
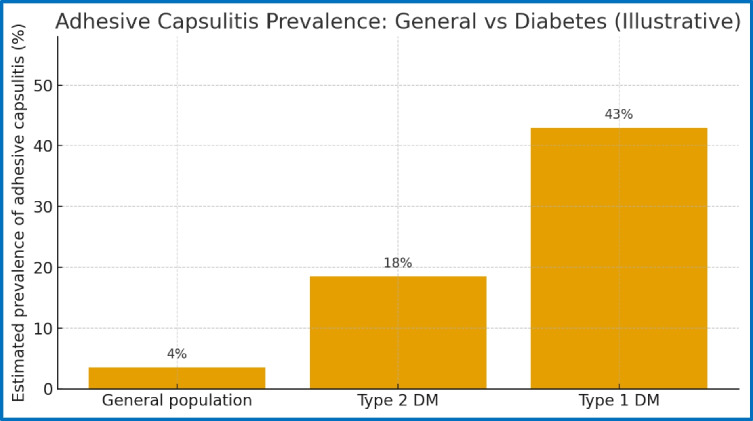
adhesive capsulitis in three different populations (DM diabetes mellitus)

Both type 1 diabetes mellitus (T1DM) and type 2 diabetes mellitus (T2DM) are linked to a higher risk of adhesive capsulitis. However, the epidemiological patterns slightly differ between the two conditions (Fig. 5).

In individuals with type 1 diabetes, reported prevalence rates vary widely, typically ranging from 10 to 76%. This variability may be related to smaller study populations, longer disease duration, and, in people with type 1 diabetes, reported prevalence rates that vary widely, usually from 10 to 76%. This variation may be linked to smaller study groups, longer disease duration, and earlier development of metabolic problems in these patients.

In contrast, in individuals with type 2 diabetes, prevalence estimates are generally lower but remain substantially higher than in the general population, typically ranging from 7 to 30%. Because type 2 diabetes is far more prevalent worldwide, it likely accounts for the majority of cases of diabetic adhesive capsulitis.

Recent Mendelian randomization studies [33–35] have suggested a potential causal relationship between type 1 diabetes and adhesive capsulitis, whereas the association with type 2 diabetes appears to be more strongly influenced by metabolic and vascular factors.

### Evidence from large cohort studies

Several large population-based studies have provided strong epidemiological evidence supporting the connection between diabetes and frozen shoulder. For example, a large cohort study [18] using UK primary care electronic health records included nearly 88,000 participants and compared individuals newly diagnosed with type 2 diabetes to matched controls without diabetes. The results showed that patients with type 2 diabetes had a significantly higher risk of developing adhesive capsulitis, with a hazard ratio of 4.38 (95% CI 3.70–5.21). During the follow-up period, 1.81% of individuals with diabetes developed adhesive capsulitis, compared to 0.63% of individuals without diabetes, highlighting the considerable effect of diabetes on shoulder health.

### Laterality and bilateral involvement

Adhesive capsulitis most often affects one shoulder, but bilateral involvement is not uncommon, especially among patients with diabetes [20]. Previous studies indicate that up to 17% of diabetic patients with adhesive capsulitis develop bilateral disease, a rate that seems higher than in non-diabetic populations.

This increased rate of bilateral involvement supports the hypothesis that systemic metabolic abnormalities associated with diabetes, rather than purely local mechanical factors, contribute to the pathogenesis of the condition.

### Geographic and ethnic variations

Epidemiological studies from Asia [36, 37] have shown higher rates of adhesive capsulitis in diabetic groups compared to Western populations. However, differences in study design, diagnostic criteria, and access to healthcare make direct comparisons challenging. Ethnic differences might also contribute, but evidence is still limited.

The association between diabetes and frozen shoulder has also been supported by several population-based studies conducted in East Asia. For example, a longitudinal population-based cohort study in Taiwan reported that patients with diabetes had a significantly higher incidence of adhesive capsulitis compared with non-diabetic individuals [16]. Similarly, another nationwide cohort study found that both diabetes and prediabetes were associated with an increased risk of developing frozen shoulder, suggesting that impaired glucose metabolism may play a role in the pathogenesis of the disease even before diabetes is fully established [21].

The strong and consistent association observed across multiple studies suggests that diabetes is not merely a coincidental comorbidity but may play a causal role in the development of the condition. Screening for shoulder stiffness in diabetic patients, especially those with long-standing or poorly controlled disease, should therefore be part of routine clinical practice [38].

## ASSOCIATED RISK FACTORS

The mechanisms by which diabetes leads to the development of frozen shoulder are not fully understood, but several biological processes (Table 3) have been suggested. One of the most widely accepted mechanisms involves the formation of advanced glycation end products (AGEs) caused by chronic hyperglycemia. Emphasis is placed on diabetes mellitus itself, as well as metabolic syndrome components such as obesity, hyperlipidemia, and hypertension, along with endocrine disorders like thyroid dysfunction, and demographic and lifestyle factors that may contribute to the disease.

**Table 3:** Risk Factors for Adhesive Capsulitis in Diabetes

Risk Factor	Role
Type 2 diabetes	Strong independent causal factor
Poor glycemic control	Promotes collagen glycation
Obesity	Chronic inflammation
Hyperlipidaemia	Metabolic inflammation
Hypertension	Vascular impairment
Thyroid dysfunction	Collagen metabolism changes
Age (40–65)	Degenerative changes
Female sex	Hormonal/autoimmune influences
Smoking/alcohol	Microvascular and inflammatory effects

### Glycaemic control

Poor glycaemic control has consistently emerged as a major determinant of adhesive capsulitis risk and severity. Elevated HbA1c levels correlate with greater incidence, more prolonged disease course, and reduced response to both conservative and surgical interventions. In individuals with poorly controlled diabetes, persistent hyperglycaemia leads to non-enzymatic glycation of collagen and other structural proteins. The accumulation of AGEs promotes collagen cross-linking in connective tissues, resulting in increased capsule thickness and stiffness and reduced joint capsule elasticity. This process contributes to capsular thickening and fibrosis, which are hallmark features of adhesive capsulitis [39].

Another proposed mechanism involves chronic low-grade inflammation associated with diabetes. Inflammatory cytokines such as interleukin-1, interleukin-6, and tumour necrosis factor-alpha are elevated in individuals with metabolic disorders and may initiate inflammatory processes within the shoulder capsule. This inflammatory response can trigger fibroblast proliferation and excessive collagen deposition, leading to progressive capsular fibrosis and joint movement restriction.

Microvascular changes associated with diabetes may also contribute to the development of frozen shoulder. Diabetes is known to cause microangiopathy, which reduces blood supply to connective tissues and impairs tissue repair processes. Reduced vascular perfusion of the shoulder capsule may promote hypoxia and fibrosis, further exacerbating capsular contracture. The coexistence of microvascular complications—retinopathy, nephropathy, and neuropathy—has been linked with higher incidence of adhesive capsulitis.

A longer disease duration is strongly associated with a higher prevalence of adhesive capsulitis. Patients with more than ten years of diabetes demonstrate significantly increased odds compared with those with recent diagnoses.

This cumulative exposure effect likely reflects chronicity of hyperglycemia, accumulation of AGEs, and progressive microvascular damage. Importantly, adhesive capsulitis may even precede the diagnosis of diabetes in some cases, suggesting that pre-diabetes and prolonged insulin resistance also contribute to risk.

### Metabolic syndrome and related risk factors

Metabolic syndrome is a cluster of metabolic abnormalities that includes obesity, hyperlipidaemia, hypertension, and insulin resistance. These conditions frequently coexist with diabetes and have been investigated as potential contributors to frozen shoulder.

Several observational studies have reported a higher prevalence of metabolic syndrome among individuals with adhesive capsulitis. For instance, studies examining serum lipid profiles have found that patients with frozen shoulder often exhibit elevated cholesterol and triglyceride levels compared with healthy controls. Hyperlipidaemia may contribute to connective tissue degeneration and inflammatory processes within the joint capsule, thereby increasing susceptibility to frozen shoulder [40].

Obesity has also been identified as a potential risk factor. Excess adipose tissue is associated with chronic low-grade inflammation and increased production of pro-inflammatory cytokines. These inflammatory mediators may influence the structure and function of connective tissues, potentially contributing to capsular fibrosis and joint stiffness.

Hypertension has likewise been investigated as a potential risk factor for frozen shoulder. Some researchers have proposed that vascular changes associated with hypertension may impair blood flow to the shoulder capsule, leading to ischaemia and subsequent fibrosis.

However, the extent to which metabolic syndrome mediates the relationship between diabetes and frozen shoulder remains uncertain. A recent cohort study examined how metabolic health—defined by the development of obesity, hyperlipidaemia [17], or hypertension—mediates the effect of type 2 diabetes on frozen shoulder risk.

### Thyroid dysfunction

Thyroid disorders represent another endocrine condition that has been linked to frozen shoulder. Both hypothyroidism and hyperthyroidism [24] have been associated with an increased risk of adhesive capsulitis, although hypothyroidism appears to be more commonly reported.

The mechanisms underlying this association are not fully understood but may involve alterations in connective tissue metabolism and autoimmune processes. Thyroid hormones play an important role in regulating collagen synthesis and degradation, and disturbances in thyroid function may disrupt the balance of these processes. In addition, autoimmune mechanisms associated with thyroid disease may contribute to inflammatory changes within the joint capsule.

Epidemiological studies have reported a higher prevalence of thyroid dysfunction among patients with frozen shoulder compared with the general population. The coexistence of thyroid disease and diabetes may further increase the risk of developing musculoskeletal complications.

### Demographic factors

Several demographic factors have been associated with an increased risk of frozen shoulder. Age is one of the most important factors, with the condition most commonly occurring between the ages of 40 and 65 years. Degenerative changes in connective tissues and reduced vascularity associated with aging may contribute to the development of capsular fibrosis and joint stiffness.

Sex differences have also been observed in the epidemiology of frozen shoulder. Many studies report a higher prevalence of the condition among women compared with men. Although the reasons for this difference are not fully understood, hormonal factors, autoimmune predisposition, and differences in connective tissue structure may play a role.

### Lifestyle factors

Lifestyle factors like smoking and alcohol use are also considered potential contributors to frozen shoulder. Smoking is known to impair microvascular circulation and tissue healing, which may worsen connective tissue damage and fibrosis. Additionally, smoking has been linked to increased systemic inflammation, which could further contribute to musculoskeletal disorders.

**Fig. 6:**
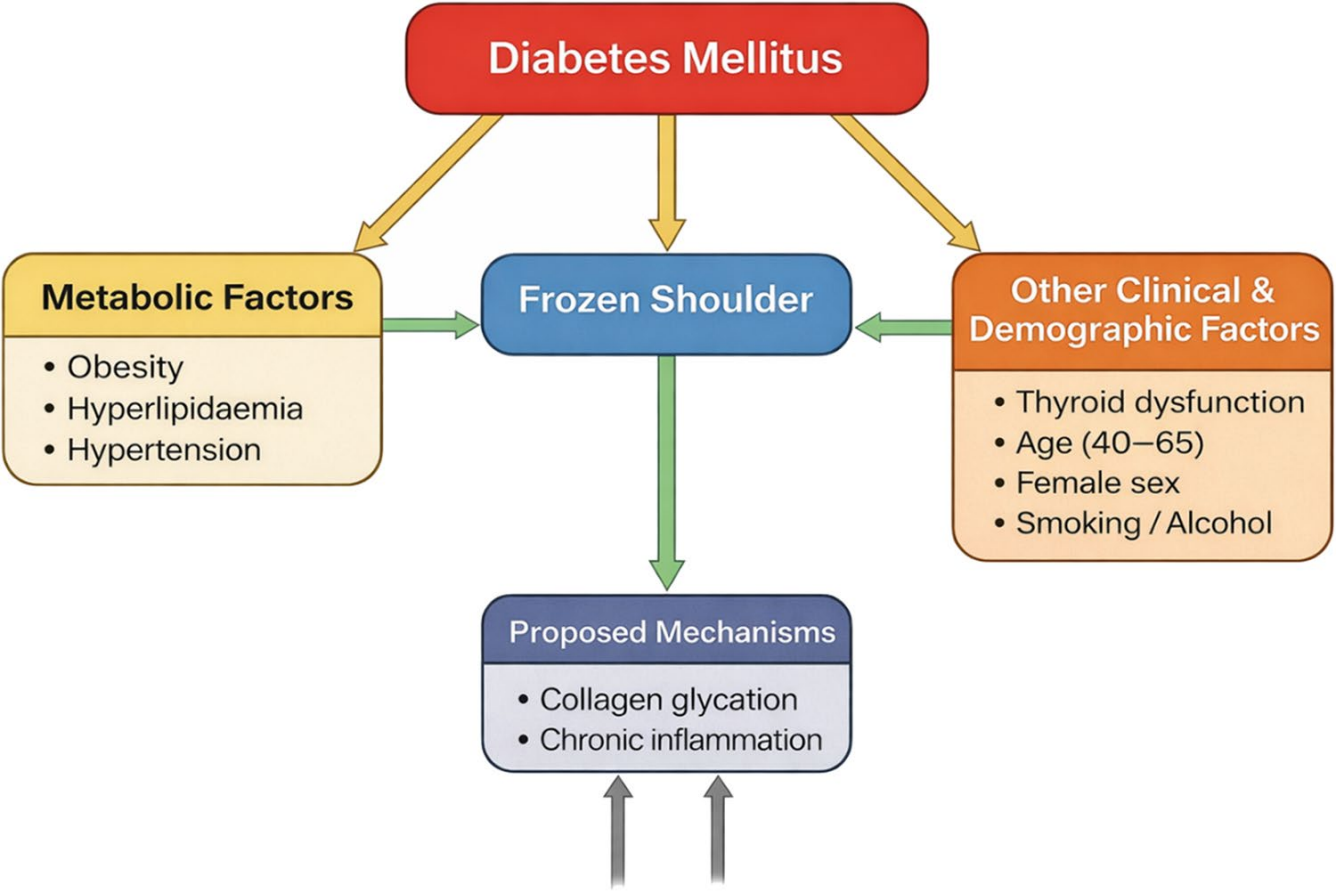
Conceptual framework for risk factors for frozen shoulder

Alcohol consumption (Fig. 6) may influence metabolic health and has been associated with various inflammatory and metabolic conditions. Although the evidence linking alcohol use directly to frozen shoulder is limited, alcohol consumption may indirectly contribute to the condition through its effects on metabolic and endocrine systems.

Physical inactivity is another potential risk factor. Limited shoulder movement or long-term immobilization can lead to capsular tightening and fibrosis. People with diabetes might be more prone to reduced physical activity due to other health issues, which could further raise their risk of developing frozen shoulder.

## Discussion

The present review confirms a strong association between diabetes mellitus and adhesive capsulitis, a relationship that has been recognized for several decades [41] but remains incompletely understood. The findings of the current analysis indicate that individuals with diabetes have a substantially increased risk of developing frozen shoulder compared with non-diabetic individuals. The pooled estimate from the meta-analysis suggests that diabetes is associated with approximately 3.7-fold higher odds of adhesive capsulitis.

These results are consistent with previous epidemiological studies and systematic reviews [42] that have reported a markedly higher prevalence of adhesive capsulitis among diabetic populations. In the general population, the prevalence of frozen shoulder is estimated to range between 2 and 5%, whereas studies in diabetic cohorts frequently report prevalence rates between 13 and 30%.

Several biological mechanisms may explain the observed association between diabetes mellitus and adhesive capsulitis. One of the most widely accepted mechanisms involves chronic hyperglycaemia, which promotes the formation of advanced glycation end products (AGEs). These molecules alter the structural properties of collagen by increasing cross-linking between collagen fibers and reducing their elasticity. As a consequence, connective tissues such as the shoulder joint capsule become progressively stiffer and less compliant. In addition, chronic hyperglycaemia may stimulate fibroblast proliferation and excessive collagen deposition, contributing to capsular thickening and fibrosis. These structural changes are consistent with histological findings observed in patients with adhesive capsulitis. Another potential mechanism involves chronic low-grade inflammation, which is commonly observed in individuals with diabetes and metabolic syndrome. Elevated levels of inflammatory cytokines may trigger inflammatory processes within the shoulder capsule, further promoting fibrosis and joint stiffness. Microvascular complications associated with diabetes may also contribute to the pathogenesis of adhesive capsulitis. Diabetes-related microangiopathy can impair blood supply to connective tissues, resulting in tissue hypoxia and impaired repair processes. These vascular alterations may further exacerbate capsular fibrosis and joint contracture.

Although diabetes mellitus appears to be a major risk factor for frozen shoulder, several metabolic and endocrine conditions frequently coexist with diabetes and may contribute to the development of adhesive capsulitis. Components of metabolic syndrome, including obesity, hyperlipidaemia, and hypertension, have been investigated as potential contributors to frozen shoulder. These conditions are associated with systemic inflammation, vascular dysfunction, and connective tissue alterations that may predispose individuals to capsular fibrosis. Thyroid disorders have also been reported more frequently in patients with adhesive capsulitis. Thyroid hormones play an important role in regulating collagen metabolism, and disturbances in thyroid function may affect connective tissue remodeling. However, current evidence suggests that these metabolic and endocrine factors do not fully explain the increased risk of adhesive capsulitis observed in diabetic populations. Instead, diabetes itself likely plays a central role in the development of the condition.

The strong association between diabetes mellitus and adhesive capsulitis has important implications for clinical practice. First, clinicians should maintain a high index of suspicion for frozen shoulder in patients with diabetes who present with shoulder pain or progressive limitation of shoulder motion. Early recognition of adhesive capsulitis may facilitate timely intervention and potentially improve functional outcomes. Second, screening for shoulder stiffness in patients with long-standing or poorly controlled diabetes may help identify cases at an earlier stage of the disease. Improved glycaemic control may theoretically reduce the risk of connective tissue changes associated with hyperglycaemia, although further research is required to determine whether optimizing metabolic control can effectively prevent the development of adhesive capsulitis. In addition, addressing modifiable risk factors, such as obesity, smoking, and physical inactivity, may contribute to reducing the overall burden of musculoskeletal complications in diabetic populations.

This study has several strengths. First, it synthesizes evidence from both cohort and case–control studies, providing a comprehensive overview of the epidemiological relationship between diabetes mellitus and adhesive capsulitis. Second, the inclusion of large population-based studies increases the generalizability of the findings. Finally, the use of a random-effects meta-analysis allows for the estimation of an overall effect size while accounting for variability between studies.

Several limitations should be considered when interpreting the findings of this review. First, the majority of the included studies were observational in design, which limits the ability to establish a causal relationship between diabetes mellitus and adhesive capsulitis. Observational studies are inherently susceptible to residual confounding, and several potential confounding variables—including age, body mass index, and physical activity—may influence the observed association. Second, there was heterogeneity in diagnostic criteria used across studies for both diabetes mellitus and adhesive capsulitis. Differences in study populations, healthcare systems, and data sources may also contribute to variability in reported prevalence and risk estimates. Third, the number of studies included in the meta-analysis was relatively limited, which restricted the ability to perform more detailed analyses such as subgroup analyses or formal tests for publication bias. Finally, most studies relied on administrative databases or clinical coding systems to identify cases of adhesive capsulitis, which may introduce misclassification bias. Although the association between diabetes mellitus and adhesive capsulitis appears strong, causality cannot be definitively established because most available evidence comes from observational studies. Residual confounding factors, such as age, body mass index, physical activity, and components of metabolic syndrome, may partially explain the observed association.

## Conclusion

This systematic review and meta-analysis demonstrate a strong epidemiological association between diabetes mellitus and adhesive capsulitis. Individuals with diabetes appear to have approximately three to four times higher odds of developing frozen shoulder compared with non-diabetic individuals. Although the underlying mechanisms remain incompletely understood, chronic hyperglycaemia, collagen glycation, inflammation, and microvascular alterations likely contribute to capsular fibrosis. Given the global rise in diabetes prevalence, increased awareness of diabetic musculoskeletal complications is essential. Future prospective studies are needed to clarify the causal mechanisms and to determine whether improved metabolic control can reduce the risk of adhesive capsulitis.

## Data Availability

No datasets were generated or analysed during the current study.

## Declarations

This study was performed in line with the principles of the Declaration of Helsinki. Informed consent was waived due to the review design of the study.
